# Reliability of cerebral autoregulation using different measures of perfusion pressure in patients with subarachnoid hemorrhage

**DOI:** 10.14814/phy2.15203

**Published:** 2022-03-28

**Authors:** Markus Harboe Olsen, Tenna Capion, Christian Gunge Riberholt, Søren Bache, Ronan M. G. Berg, Kirsten Møller

**Affiliations:** ^1^ Department of Neuroanaesthesiology The Neuroscience Centre Copenhagen University Hospital ‐ Rigshospitalet Copenhagen Denmark; ^2^ Department of Neurosurgery The Neuroscience Centre Copenhagen University Hospital ‐ Rigshospitalet Copenhagen Denmark; ^3^ Department of Neurorehabilitation/Traumatic Brain Injury Unit The Neuroscience Centre Copenhagen University Hospital ‐ Rigshospitalet Copenhagen Denmark; ^4^ Department of Clinical Physiology and Nuclear Medicine Copenhagen University Hospital ‐ Rigshospitalet Copenhagen Denmark; ^5^ Centre for Physical Activity Research Rigshospitalet Copenhagen University Hospital Copenhagen Denmark; ^6^ Department of Biomedical Sciences Faculty of Health and Medical Sciences University of Copenhagen Copenhagen Denmark; ^7^ Neurovascular Research Laboratory Faculty of Life Sciences and Education University of South Wales Pontypridd United Kingdom; ^8^ Institute of Clinical Medicine Faculty of Health and Medical Sciences University of Copenhagen Copenhagen Denmark

**Keywords:** autoregulation, mean flow index, Mx, reliability, transfer function analysis

## Abstract

Dynamic cerebral autoregulation to spontaneous fluctuations in cerebral perfusion pressure (CPP) is often assessed by transcranial Doppler (TCD) in the time domain, yielding primarily the mean flow index (Mx), or in the frequency domain using transfer function analysis (TFA), yielding gain and phase. For both domains, the measurement of blood pressure is critical. This study assessed the inter‐method reliability of dynamic cerebral autoregulation using three different methods of pressure measurement. In 39 patients with aneurysmal subarachnoid hemorrhage, non‐invasive arterial blood pressure (ABP), invasive ABP (measured in the radial artery) and CPP were recorded simultaneously with TCD. Intraclass correlation coefficient (ICC) was used to quantify reliability. Mx was higher when calculated using invasive ABP (0.39; 95% confidence interval [95% CI]: 0.33; 0.44) compared to non‐invasive ABP, and CPP. The overall ICC showed poor to good reliability (0.65; 95% CI: 0.11; 0.84; *n* = 69). In the low frequency domain, the comparison between invasively measured ABP and CPP showed good to excellent (normalized gain, ICC: 0.87, 95CI: 0.81; 0.91; *n* = 96; non‐normalized gain: 0.89, 95% CI: 0.84; 0.92; *n* = 96) and moderate to good reliability (phase, ICC: 0.69, 95% CI: 0.55; 0.79; *n* = 96), respectively. Different methods for pressure measurement in the assessment of dynamic cerebral autoregulation yield different results and cannot be used interchangeably.

## Graphical abstract

Mx calculated using invasive arterial blood pressure (ABP) (Mxa) results in higher values than those calculated using non‐invasive ABP (nMxa) and cerebral perfusion pressure (CPP) (Mxc). The comparison between Mxa, nMxa, and Mxc showed moderate reliability at best. The gain and phase in the low‐frequency domain range for transfer function analysis (TFA) showed good reliability when comparing TFA measured using CPP and invasive ABP. We advise against using these measures interchangeably.

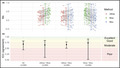

## 1 INTRODUCTION

Acute fluctuations in cerebral perfusion pressure (CPP) challenge the requirement for a constant cerebral blood flow. Dynamic cerebral autoregulation dampens these changes by adjusting the cerebrovascular resistance and can be assessed in humans through several (most commonly) transcranial Doppler ultrasound (TCD)‐based methods (Claassen et al., 2021). Many of these involve CPP measurements, which necessitates the measurement of intracranial pressure (ICP) and arterial blood pressure (ABP). The latter has often been measured either invasively or non‐invasively as a surrogate of CPP (Claassen et al., 2015; Olsen et al., 2022; Petersen et al., 2014).

Results from studies using different pressure measurements are readily compared in the literature, even though the influence of the chosen method for the measurement of perfusion pressure is not fully understood (Olsen et al., 2022). Dynamic cerebral autoregulation to spontaneous CPP fluctuations may be assessed in either the time domain investigating changes in signal over time, yielding measures such as the mean flow index (Mx) (Czosnyka et al., 1996), or in the frequency domain investigating the distribution of the signal within different frequency bands, often by transfer function analysis (TFA), which yields the metrics gain, phase, and coherence (Claassen et al., 2015).

In the present study, we sought to assess the reliability of Mx and TFA when using non‐invasive ABP, invasive ABP, and CPP (calculated by subtracting ICP from invasive ABP) in patients with aneurysmal subarachnoid hemorrhage (SAH).

## 2 METHODS

The present work was a prospective controlled intervention study designed to evaluate the effects of noradrenaline‐induced hypertension in patients admitted to the neurointensive care unit (neuro‐ICU) with SAH. This work comprised exploratory analyses of selected data and addressed an independent working hypothesis. The study was registered at clinicaltrials.gov (NCT03987139, June 14, 2019) and approved by the Regional Ethical Committee of the Capital Region of Denmark (H‐19017185, May 28, 2019), and by The Danish Data Protection Agency (P‐2019‐87, May 14, 2019). Oral and written informed consent was obtained from the next‐of‐kin. The data underlying the findings of this study can be shared upon reasonable request and only after approval from the corresponding author and relevant regulatory authorities.

### 2.1 Subjects and recordings

Adults (≥18 years old) admitted to the neuro‐ICU with SAH and treated with an external ventricular drain were eligible for inclusion. The exclusion criteria were conservative treatment of the aneurysm, expected death within 48 h from admission, and acute or chronic diseases associated with impaired cerebral autoregulation (e.g., previous ischaemic stroke, sepsis within a year before admission, diabetes mellitus with organ manifestation, or traumatic brain injury). For inclusion in the present study, at least one autoregulation measurement, either baseline or induced hypertension, with simultaneous recording for more than 5 min of at least two of the three pressure measurements (i.e., non‐invasive ABP, invasive ABP or CPP [calculated by subtracting ICP from invasive ABP]) was required. This study included recordings from 40 participants. Subject and recording characteristics are provided in Table 1. The study was performed as a before‐and‐after study with two repeated autoregulation assessments, and both recordings were included for this study if they met the above‐mentioned requirements.

**TABLE 1:** Study characteristics

Participants (*n* = 40)	
Age (years) – median (IQR)	58 (51–64)
Male – *n* (%)	8 (20%)
Poor‐grade SAH (WFNS 4–5) – *n* (%)	28 (70%)
Heart rate (min^−1^) – mean ± SD	79.0 ± 23.6
Middle cerebral artery velocity (cm/s) – mean ± SD	65.7 ± 25.5
Mean flow index (Mx)	
Recordings – *n*	95
Recording length baseline (min) – median (IQR)	27.3 (19.3–30.0)
Recording length induced hypertension (min) – median (IQR)	23.0 (17.0–27.6)
Artifacts (%) – median (IQR)	0.06 (0–0.13)
TFA	
Recordings – *n*	99
Recording length, baseline (min) – median (IQR)	24.5 (15.6–28.1)
Recording length, induced hypertension (min) – median (IQR)	19.9 (12.4–25.3)

Abbreviations: SAH, aneurysmal subarachnoid hemorrhage; TFA, transfer function analysis; WFNS, World federation of neurological surgeons.

### 2.2 Transcranial Doppler

For each TCD session, an insonation probe (DWL) was kept stable by a LAM rack (DWL). There were no changes to the TCD settings, and the patients did not change position during recordings. Middle cerebral artery flow velocity was measured through the ipsilateral transtemporal window using MultiDop T digital (DWL) (Newell & Aaslid, 1992). ICP was measured using either a Codman Microsensor ICP Transducer (Integra LifeSciences) or a Spiegelberg external ventricular drain combined with an ICP sensor (Spiegelberg). ABP was measured invasively through a radial artery catheter and/or non‐invasively by photoplethysmography (Nano System, ADInstruments Inc.). The recordings were synchronzed using an analog‐to‐digital converter from AD Instruments and synchronized in LabChart (LabChart ver. 8.10.05, ADInstruments Inc.).

### 2.3 Data processing

#### 2.3.1 Calculation of Mx

Raw waveform data of TCD, ICP, and ABP were extracted from LabChart into a tab‐delimited file in the original resolution of 1000 Hz. The recordings were visually inspected for artifacts. Artifacts in any recording modality resulted in the removal of a period surrounding that artifact, always starting and ending with a curve nadir for the specific recording modality. Mx was calculated by averaging waveform data into blocks, which were then grouped into epochs, where a correlation coefficient was calculated. All the correlation coefficients were then averaged into one Mx‐value for the full recording period. To ensure sufficient quality of the calculations, blocks were omitted from the analysis if more than 50% of the raw measurements were missing, and epochs were omitted if more than 50% of the blocks were missing. Mx was calculated using the ‘*clinmon*’‐function from the publicly available R package ‘clintools’ v. 0.8.2 (Olsen et al., 2021). We have validated the ‘*clinmon*’‐function by comparing the results when calculating Mx using the ICM+ software which was used to develop Mx. This validation shown nearly perfect reliability (intraclass correlation coefficient [ICC]‐agreement: 1.00 [95% CI: 0.98; 1.00]; Recordings compared = 76; data not shown).

#### 2.3.2 Calculation of TFA metrics

Raw waveform data were averaged using the ‘cyclic measurement’‐function in Labchart and extracted in the original resolution of 1000 Hz. The recordings were visually inspected for artifacts; only continuous periods without any artifacts were extracted in order to avoid interpolation as a potential confounder (Claassen et al., 2015), resulting in shorter recording periods. The TFA metrics were calculated using the ‘*TFA*’‐function from the publicly available R package ‘*clintools*’ v. 0.8.2 (Olsen et al., 2021).

We have validated the ‘*TFA*’‐function by comparing the results when calculating TFA using the publicly available MatLab‐code from David Simpson (Claassen et al., 2015). This validation showed perfect reliability (ICC‐agreement; normalized gain, non‐normalized gain, and phase in the low‐frequency domain: 1.00 [95% CI: 1.00; 1.00]; recordings compared = 53; data not shown). This script follows the recommendations including the application of a coherence threshold identified using 95% confidence limits based on degrees of freedom. The package follows the recommendation where all frequencies with low magnitude‐squared coherence are excluded from averaging when calculating the mean values of gain and phase across the bands below this threshold (Claassen et al., 2015).

### 2.4 Terminology and interpretation

#### 2.4.1 Mx

nMxa is used when ABP was measured non‐invasively, whereas Mxa is used for the invasively measured ABP and Mxc for the index calculated using CPP. Mx was interpreted as a continuous measure ranging from −1 to 1, with higher values indicating less effective cerebral autoregulation and vice versa (Czosnyka et al., 1996; Olsen et al., 2022).

#### 2.4.2 TFA

nTFA is used for non‐invasively measured ABP, TFAa for invasively measured ABP, and TFAc for CPP. Specifically, gain and phase in the low frequency (LF) range from 0.07 to 0.20 Hz (Claassen et al., 2015) were interpreted to reflect dynamic cerebral autoregulation, with higher gain and/or lower phase indicating less efficacious autoregulation and vice versa (Claassen et al., 2015; Zhang et al., 1998). Gain wil be presented as both normalized gain and non‐normalized gain.

### 2.5 Assessment of reliability

The reliability of Mx and TFA was assessed by comparing the different values based on CPP, MAP, and nMAP. For Mx, the analyses were carried out for four different approaches, which were pragmatically chosen because they were the most common approaches in the literature (Olsen et al., 2022). The primary assessment was pragmatically chosen as Mx calculated using 3‐s blocks and 60‐s epochs (3–60‐F). Exploratory assessments of Mx were 6‐s blocks and 240‐s epochs (6–240‐F), 10‐s blocks and 300‐s epochs (10–300‐F), and 10‐s blocks and 300‐s epochs with 60‐s overlaps (10–300–60). The primary assessment for the TFA metrics was normalized gain, non‐normalized gain, and phase for the LF domain.

### 2.6 Statistical analysis

All statistical analyses were carried out using R 4.1.0 (R Core Team [2021], Vienna, Austria). Normally distributed data are presented as mean (±SD), while non‐normally distributed data are presented as median (IQR). Reliability was calculated using the two‐way mixed‐effects, single measurement, absolute agreement ICC, and classified as poor (<0.5), moderate (0.5–0.75), good (0.75–0.9), or excellent (>0.9) with reference to both the lower and upper confidence limits (Koo & Li, 2016). Bland‐Altman plots were used to quantify the difference (bias) and presented with limits of agreement (LOA) (Bland & Altman, 1986). Error bars in the figures represent the 95% confidence interval (95% CI).

## 3 RESULTS

This study included 40 participants with SAH. We were able to calculate Mx for 95 periods from 39 participants (baseline: *n* = 62; induced hypertension: *n* = 33), and TFA for 99 periods from 39 participants (baseline: *n* = 64; induced hypertension: *n* = 35) (Table 1). The participants had higher invasive (mean: 88.9, 95% CI: 84.4; 93.4) than non‐invasive ABP (mean: 81.5, 95% CI: 75.7; 87.4; *p*‐value: 0.047) during baseline and comparable pressures during periods of induced hypertension (invasive ABP, mean: 93.1, 95% CI: 89.6; 96.5; non‐invasive ABP: 95.1, 95% CI: 85.2; 105.0; *p*‐value: 0.70). The ICP was 8.6 (mean; 95% CI: 7.1; 11.2) during baseline and 9.1 (95% CI: 6.7; 11.5) during periods of induced hypertension.

### 3.1 Time domain measures

**FIGURE 1:**
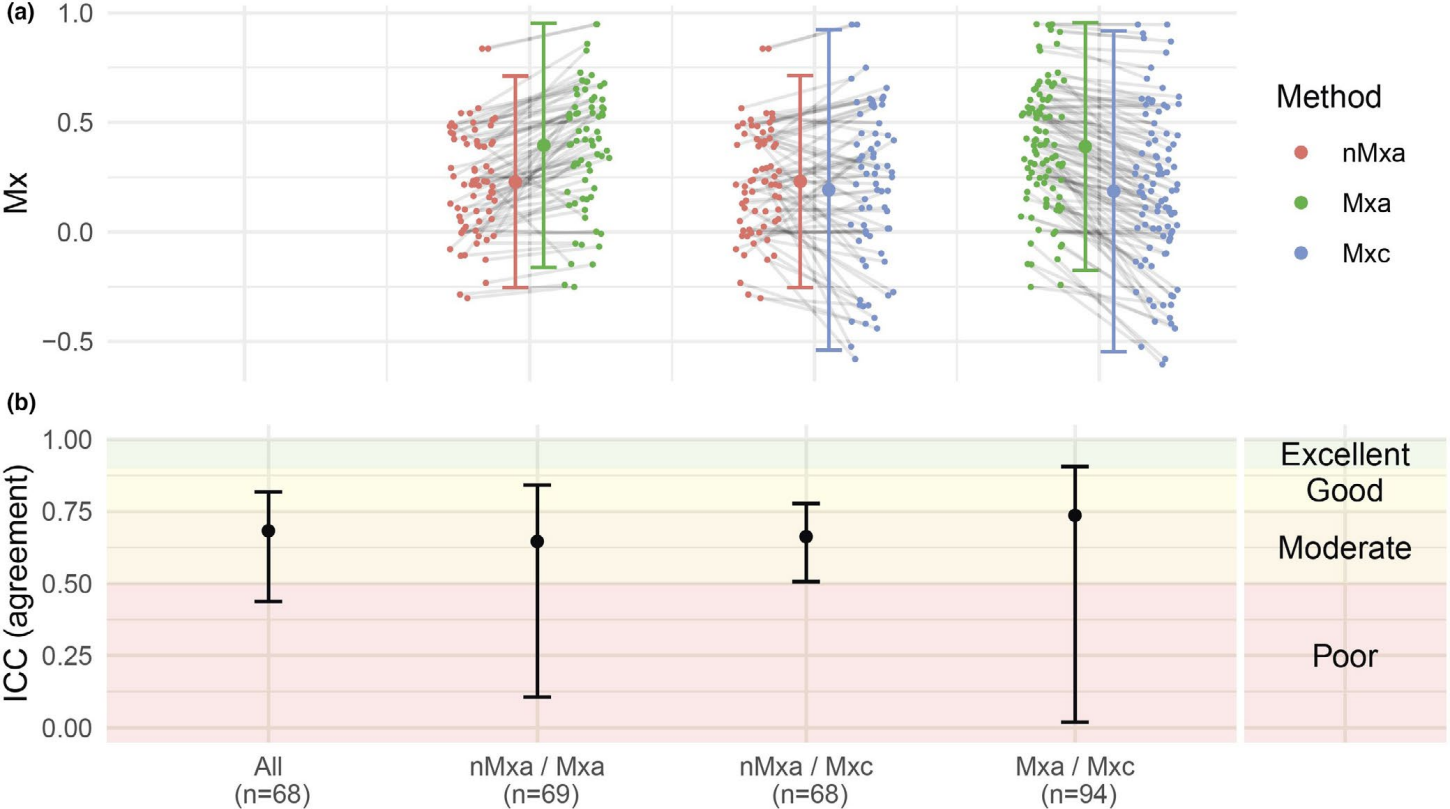
Reliability of Mx (3–60‐F) by pressure measurement. (a) Individual‐level values of Mx by pressure measurement. The grey lines depict the relationship between the results gained from the left and right approach for each comparison. Only results with corresponding measurements are presented. (b) ICC values. ICC, intraclass correlation coefficient

Mxc was 0.19 (mean; 95% CI: 0.11; 0.26), Mxa was 0.39 (95% CI: 0.33; 0.44), and nMxa was 0.23 (95% CI: 0.18; 0.28). In the Bland‐Altman plots, the smallest bias was observed between Mxc and nMxa (bias: −0.04; LOA: −0.54; 0.46), while the bias was higher for comparison between Mxc and Mxa (bias: −0.20; LOA: −0.52; 0.11) and between Mxa and nMxa (bias: 0.17; LOA: −0.17; 0.51) (Supplemental Material). The overall ICC was 0.68 (95% CI: 0.44; 0.82; *n* = 68), with similar results when comparing nMxa with Mxa (ICC: 0.65; 95% CI: 0.11; 0.84; *n* = 69), nMxa with Mxc (ICC: 0.66; 95% CI: 0.51; 0.78; *n* = 68), and Mxa with Mxc (ICC: 0.74; 95% CI: 0.02; 0.91; *n* = 94) (Figure 1). The reliability of Mx was similar irrespective of block and epoch sizes, and regardless of measurement during baseline and induced hypertension (Supplemental Material).

### 3.2 Frequency domain measures (TFA)

**FIGURE 2:**
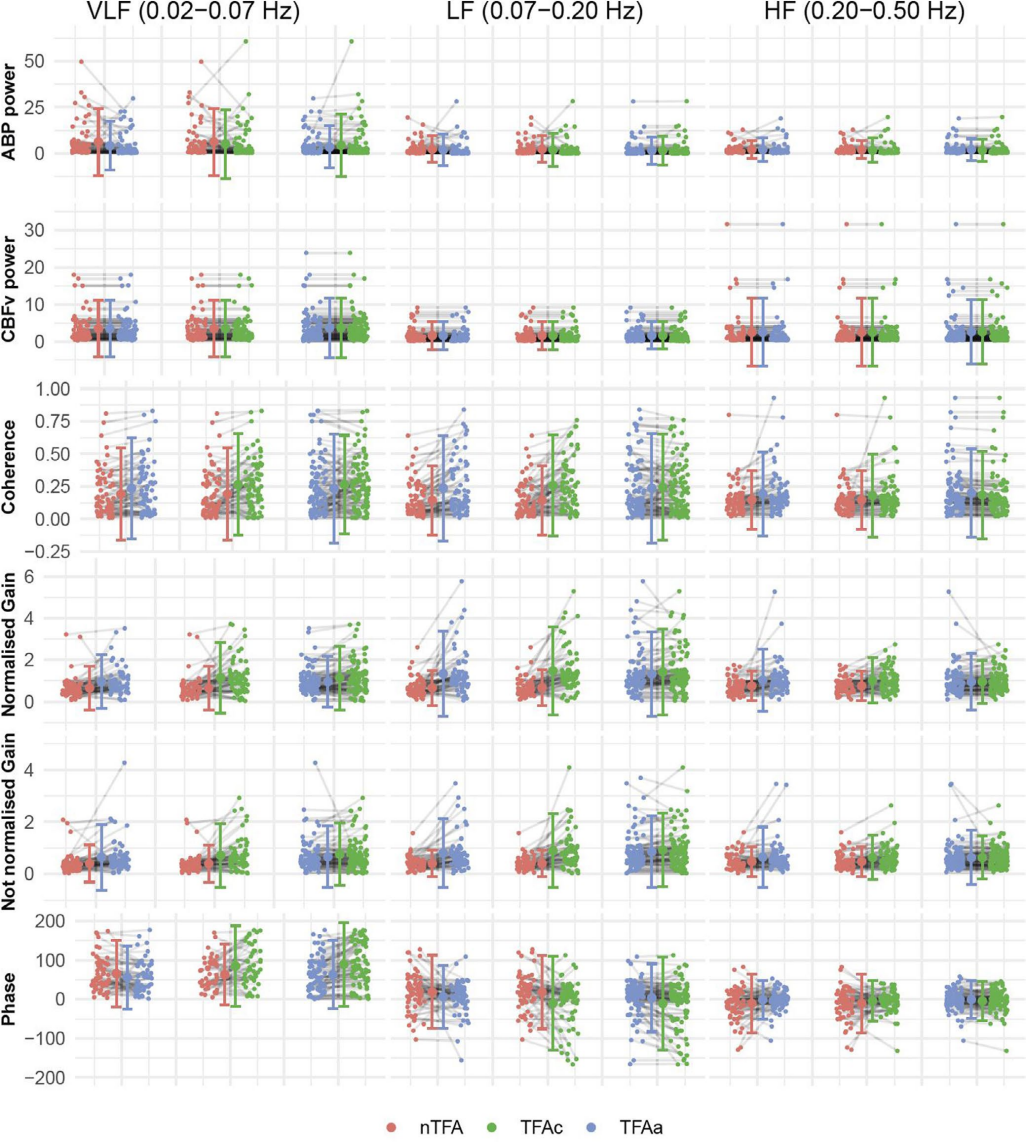
Individual‐level TFA values by pressure measurement. Grey lines depict the relationship between the results for each of the different TFA metrics obtained by the left and right approach for each comparison. Only results with corresponding measurements are presented. ABP, arterial blood pressure; CBFv, cerebral blood flow velocity; HF, high frequency; LF, low frequency; VLF, very low frequency

**FIGURE 3:**
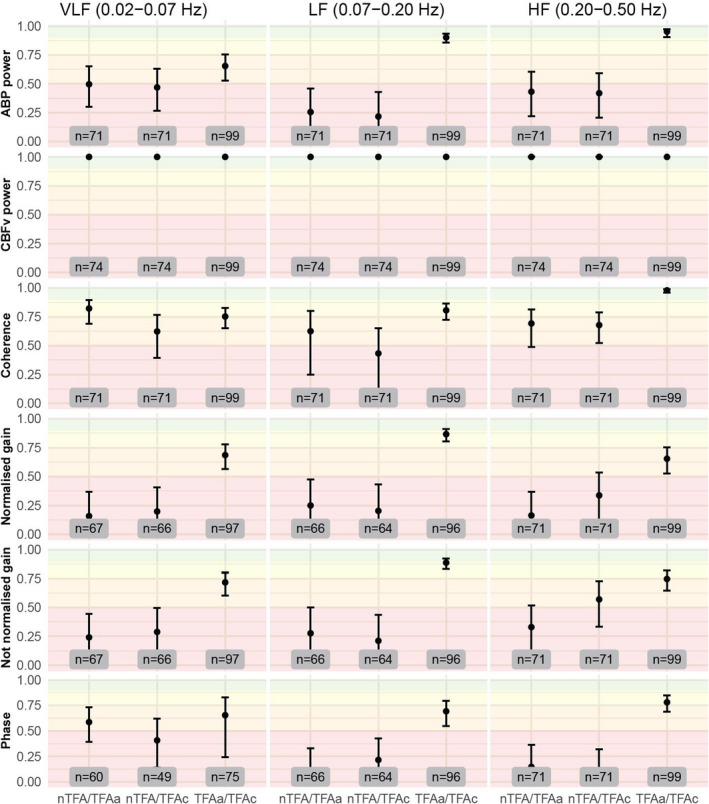
ICC by measurement approach. Green depicts the spectrum of excellent reliability, yellow of good reliability, orange of moderate reliability, and red of poor reliability. ABP, arterial blood pressure; CBFv, cerebral blood flow velocity; HF, high frequency; ICC, intraclass correlation coefficient; LF, low frequency; VLF, very low frequency

The confidence intervals of gain and phase in the LF range were wider when measured by TFAc (non‐normalized gain: 0.92, 95% CI: 0.78; 1.06; normalized gain: 1.43, 95% CI: 1.22; 1.63; phase: −9.95, 95% CI: −19.2; −0.68) and TFAa (non‐normalized gain: 0.83, 95% CI: 0.73; 0.94; normalized gain: 1.33, 95% CI: 1.17; 1.49; phase: 5.18, 95% CI: −1.36; 11.7) than by nTFA (non‐normalized gain: 0.40, 95% CI: 0.36; 0.45; normalized gain: 0.67; 95% CI: 0.60; 0.75; phase: 18.8, 95% CI: 10.6; 26.9) (Figure 2). Both normalized and non‐normalized gain in the LF range showed good to excellent reliability for the comparison between TFAc and TFAa (normalized gain: ICC: 0.87; 95% CI: 0.81; 0.91; *n* = 96; non‐normalized gain: ICC: 0.89; 95% CI: 0.84; 0.92; *n* = 96). The ICC for phase when comparing TFAc with TFAa in the LF range was 0.69 (95% CI: 0.55; 0.79; *n* = 96) (Figure 3). Overall, the smallest bias and narrowest LOA were seen in the Bland‐Altman plots when comparing TFAc with TFAa (Supplemental Material).

## 4 DISCUSSION

The present study is the first to use simultaneous measurements of non‐invasive ABP, invasive ABP, and CPP to assess the reliability of the two TCD derived parameters, Mx and TFA, for assessing dynamic cerebral autoregulation in patients with SAH.

We found that the Mx based on non‐invasive and invasive blood pressure measurements alone were higher than the mean flow index incorporating ICP; this is in line with previous reports (Liu et al., 2015; Petersen et al., 2014; Schmidt, Czosnyka, et al., 2003; Schmidt, Piechnik, et al., 2003). The overall smaller difference between nMxa and Mxa is also in line with previous reports (Lavinio et al., 2007; Petersen et al., 2014). The lower confidence limit of all Mx comparisons (i.e., nMxa, Mxa, and Mxc) of reliability was located in the range of “poor reliability”, except for the comparison between nMxa and Mxc. This raises doubts about the ability to directly compare results gained from nMxa, Mxa or Mxc. Our finding that the reliability of Mx is poor when calculated by different pressure measurements, as well as the fact that Mx in general has been reported as unreliable, unstable, highly influenced by recording length, and the choices made during preprocessing (Lorenz et al., 2007; Mahdi et al., 2017; Ortega‐Gutierrez et al., 2014; Riberholt et al., 2021), restricts the ability to collate previous literature, or even interpret the findings in relation to any physiological phenomenon.

The fact that more than 128 peer‐reviewed articles have calculated Mx and interpreted this as cerebral autoregulation is based on previous publications describing the validity of Mx (Olsen et al., 2022). Mx has been widely accepted as a measure of dynamic cerebral autoregulation partly due to its association with functional outcomes in patients with severe traumatic brain injury (Czosnyka et al., 1996), and due to its correlation with the rate of the regulation (RoR) (Lang et al., 2002; Piechnik et al., 1999), pressure reactivity index (PRx) (Lang, Lagopoulos, Griffith, Yip, Yam, et al., 2003; Pochard et al., 2020; Schmidt et al., 2012; Zeiler, Cardim, et al., 2018; Zeiler et al., 2017; Zeiler, Smielewski, et al., 2018), autoregulation index (ARI) (Czosnyka et al., 2008; Liu et al., 2020), ABP (Crippa et al., 2018), and carbon dioxide (CO_2_)‐reactivity (Zhang et al., 2016). In our opinion, almost all of these correlations have been flawed due to suspected heteroscedasticity (Gollion et al., 2019; Quispe Cornejo et al., 2020), one of the comparators being categorical (Budohoski et al., 2012; Czosnyka et al., 1996, 1997; Lang, Lagopoulos, Griffith, Yip, Mudaliar, et al., 2003; Reinhard et al., 2012; Schmidt, Lezaic, et al., 2016; Schmidt, Reinhard, et al., 2016; Tang et al., 2008), or mathematical coupling (Aggarwal & Ranganathan, 2016; Schober & Schwarte, 2018). Indices such as RoR (Lang et al., 2002; Piechnik et al., 1999), PRx (Lang, Lagopoulos, Griffith, Yip, Yam, et al., 2003; Pochard et al., 2020; Schmidt et al., 2012; Zeiler, Cardim, et al., 2018; Zeiler et al., 2017; Zeiler, Smielewski, et al., 2018), ARI (Czosnyka et al., 2008; Liu et al., 2020), ABP (Crippa et al., 2018), and CO_2_ reactivity (Zhang et al., 2016) all use the same data, increasing the risk that the correlation identified might be caused by the interdependency of data, rather than physiological associations (Aggarwal & Ranganathan, 2016; Schober & Schwarte, 2018).

In contrast, the phase and gain in the low‐frequency range in this material (normalized and non‐normalized) showed higher reliability for the comparison of invasively measured ABP with CPP. The ICC for these comparisons ranged from good to excellent. However, comparison with non‐invasively measured ABP yielded confidence limits in the area of poor reliability. Even though TFA metrics calculated using invasively measured ABP and CPP are comparable, TFA has its limitations. Thus, so far TFA has been reported to have poor repeatability and reproducibility, has been assigned no reference values, and furthermore has limited diagnostic usefulness (Chi et al., 2018; Claassen et al., 2015; Lorenz et al., 2007; Ortega‐Gutierrez et al., 2014; Sanders et al., 2018). Whether TFA can predict outcomes more precisely than Mx needs further investigation.

As a methodological curiosity, it might be important to notice the generally high ICC for the TFA metrics when comparing TFAc and TFAa in all frequency ranges. Both methods use the invasive ABP as a factor in the analysis, as the CPP is calculated by subtracting ICP from invasive ABP. If ICP is stable during measurements, this would explain the similar gain and phase values; however, the reason for the more tenuous ICC values when comparing Mxa and Mxc remains elusive.

### 4.1 Strengths and limitations

This study applied the different methods for blood pressure measurement simultaneously, eliminating the risk of time‐course effects between methods. A difference between invasive and non‐invasive ABP is comparable with previous reports (Kim et al., 2014). The open‐source preprocessing (Olsen et al., 2021) with a validated script ensured optimal transparency. A large sample size, with recording lengths above the minimum recommended, was included (Mahdi et al., 2017; Olsen et al., 2022). The lack of a global standard bedside measure of cerebral autoregulation renders us unable to select one of the pressure measurement methods as the best. As the most significant limitation, we were not able to record all three pressure measurements in every patient. Moreover, the study investigated the reliability in patients with SAH which might restrict extrapolation of the results to patients with other types of acute brain injury. Finally, the ICC in itself is an arbitrary measure of reliability. It is highly dependent on how data are dispersed, where an outcome represented on a large scale with large variation will in general yield a higher ICC value than values with only a small dispersion (Müller & Büttner, 1994). This might be the case for some of the analysis in our SAH cohort and is, therefore, a limitation of ICC on the entire scale.

## 5 CONCLUSION

According to this study in patients with aneurysmal SAH, simultaneously measured non‐invasive ABP, invasive ABP and CPP yields different results when calculating Mx or TFA measures for the evaluation of dynamic cerebral autoregulation. The reliability for the comparison of Mx measures was moderate at best, while the gain and phase in the low‐frequency domain for TFA showed good reliability. We advise against using these measures interchangeably. Whether TFA is a better method than Mx for quantifying dynamic cerebral autoregulation needs further investigation.

## CONFLICT OF INTERESTS

No conflicts of interest or competing interests were reported by the authors.

## AUTHOR CONTRIBUTIONS

Markus Harboe Olsen, Ronan M. G. Berg, Søren Bache, and Kirsten Møller designed the study; Markus Harboe Olsen did the interventions; Markus Harboe Olsen and Tenna Capion collected the data; Markus Harboe Olsen and Christian Gunge Riberholt handled and analyzed the data; All authors critically revised and accepted the final draft for publication.

## ETHICS APPROVAL

This physiological study was approved by the Regional Committee on Health Research Ethics in the Capital Region in Denmark (H‐19017185; May 28, 2019).

## CONSENT TO PARTICIPATE

Informed consent was obtained by the next‐of‐kin.

## Supporting information



Supplementary Material

## Data Availability

Data will be made available upon reasonable request to the corresponding author, and provided regulatory approvals are obtained.
