# Supplementary material for: Reliability of cerebral autoregulation using different measures of perfusion pressure in patients with subarachnoid hemorrhage
Source: Physiol Rep. 2022 Mar 28;10(6):e15203. doi: 10.14814/phy2.15203 (PMC8958499; doi:10.14814/phy2.15203)

Statistical report for

Reliability study

25 januar 2022

Contents

Mx with other block- and epoch sizes. **2**

Supplemental Figure 0 - Mx (3-60-F) 2

Supplemental Figure 1 - Mx (6-240-F) 2

Supplemental Figure 2 - Mx (10-300-F) 3

Supplemental Figure 3 - Mx (10-300-60) 4

Reliability of specific periods **5**

Supplemental Figure 4 - Mx for baseline (3-60-F) 5

Supplemental Figure 5 - Mx for induced hypertension (3-60-F) 6

Bland-Altman plot for TFA **7**

Mx with other block- and epoch sizes.

Supplemental Figure 0 - Mx (3-60-F)

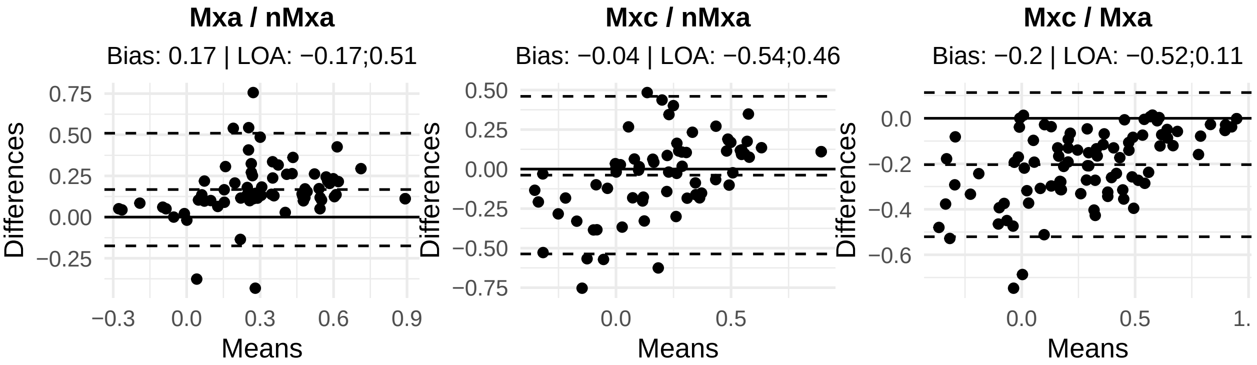

Supplemental Figure 1 - Mx (6-240-F)

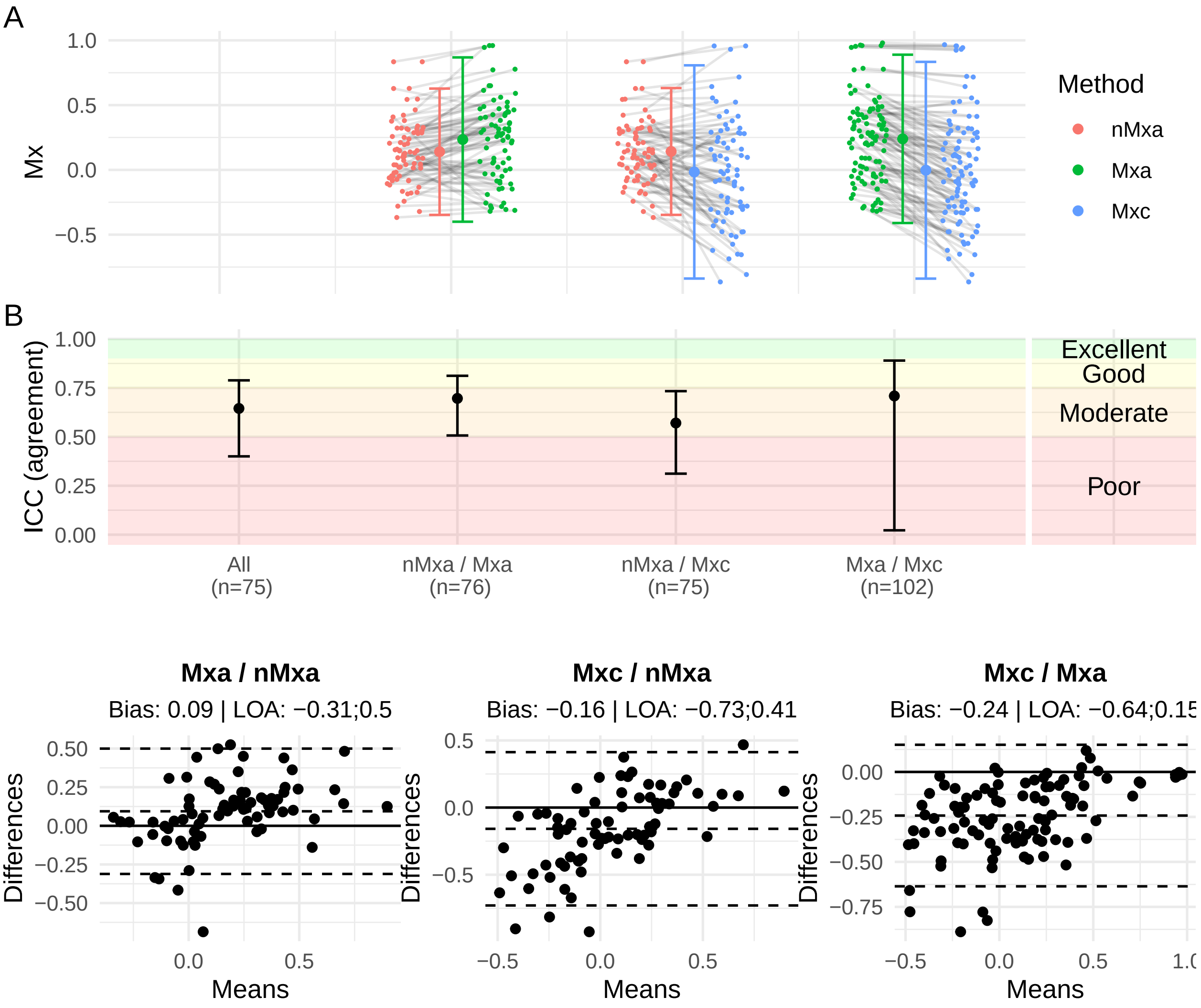

Supplemental Figure 2 - Mx (10-300-F)

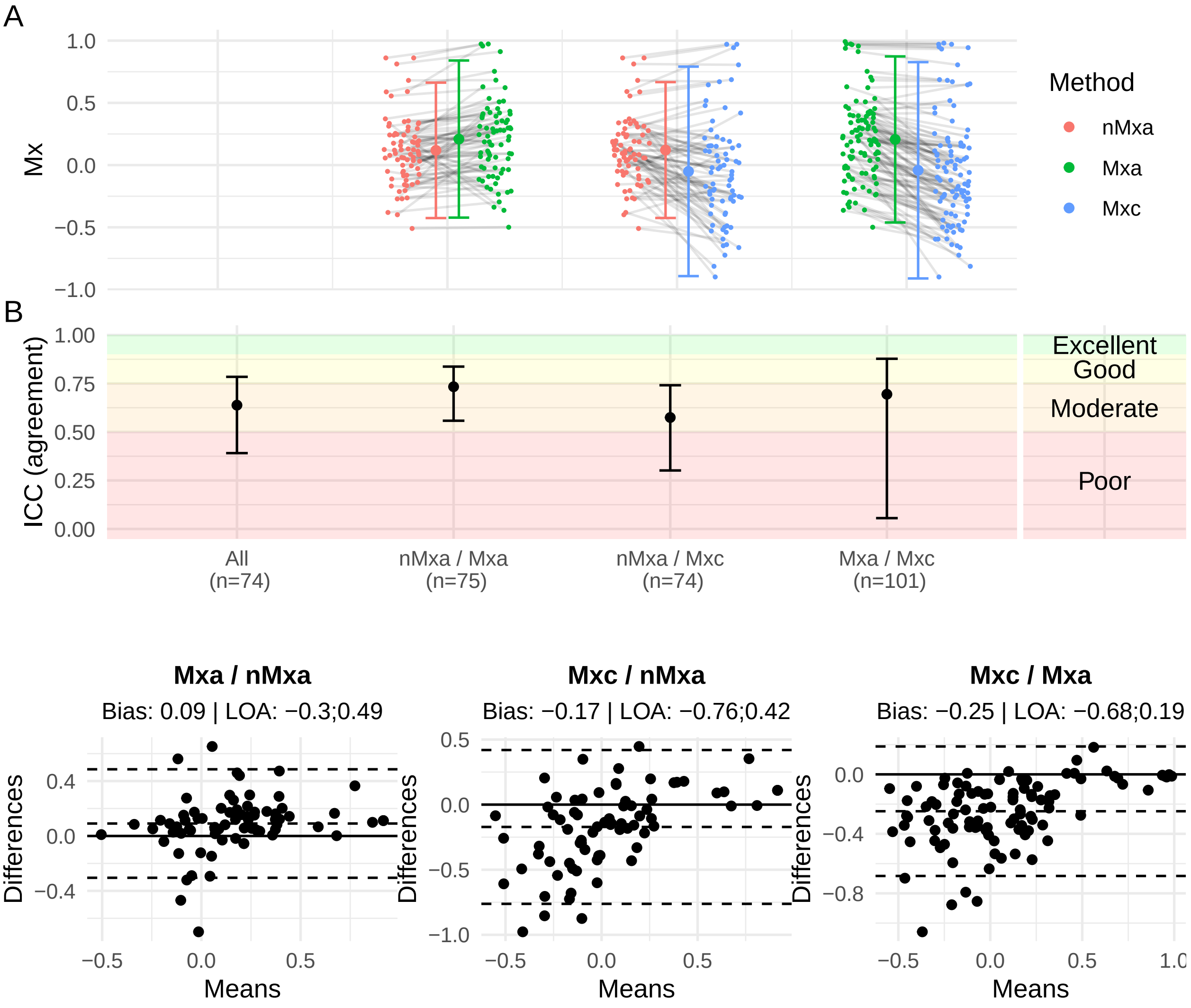

Supplemental Figure 3 - Mx (10-300-60)

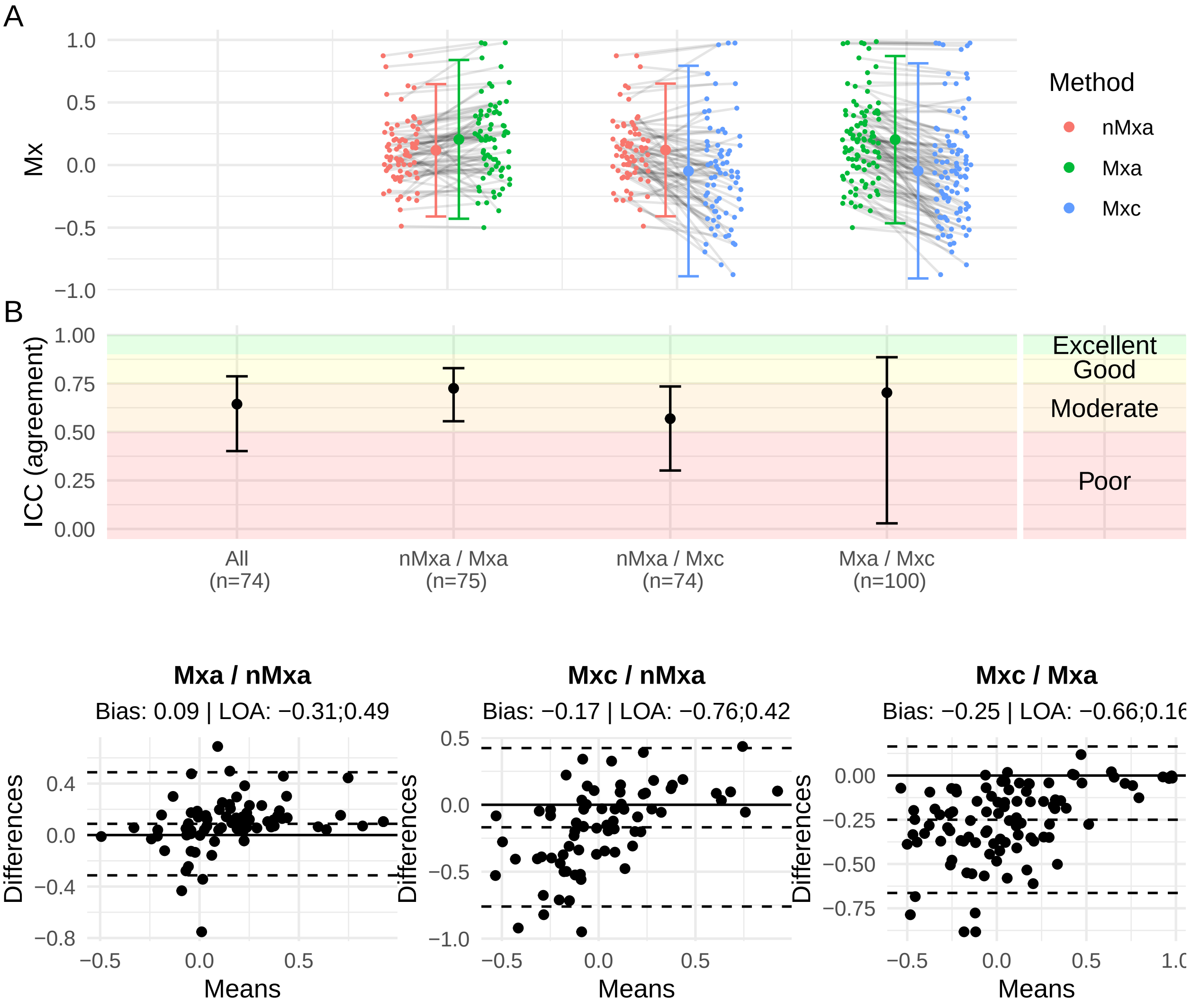

Reliability of specific periods

Supplemental Figure 4 - Mx for baseline (3-60-F)

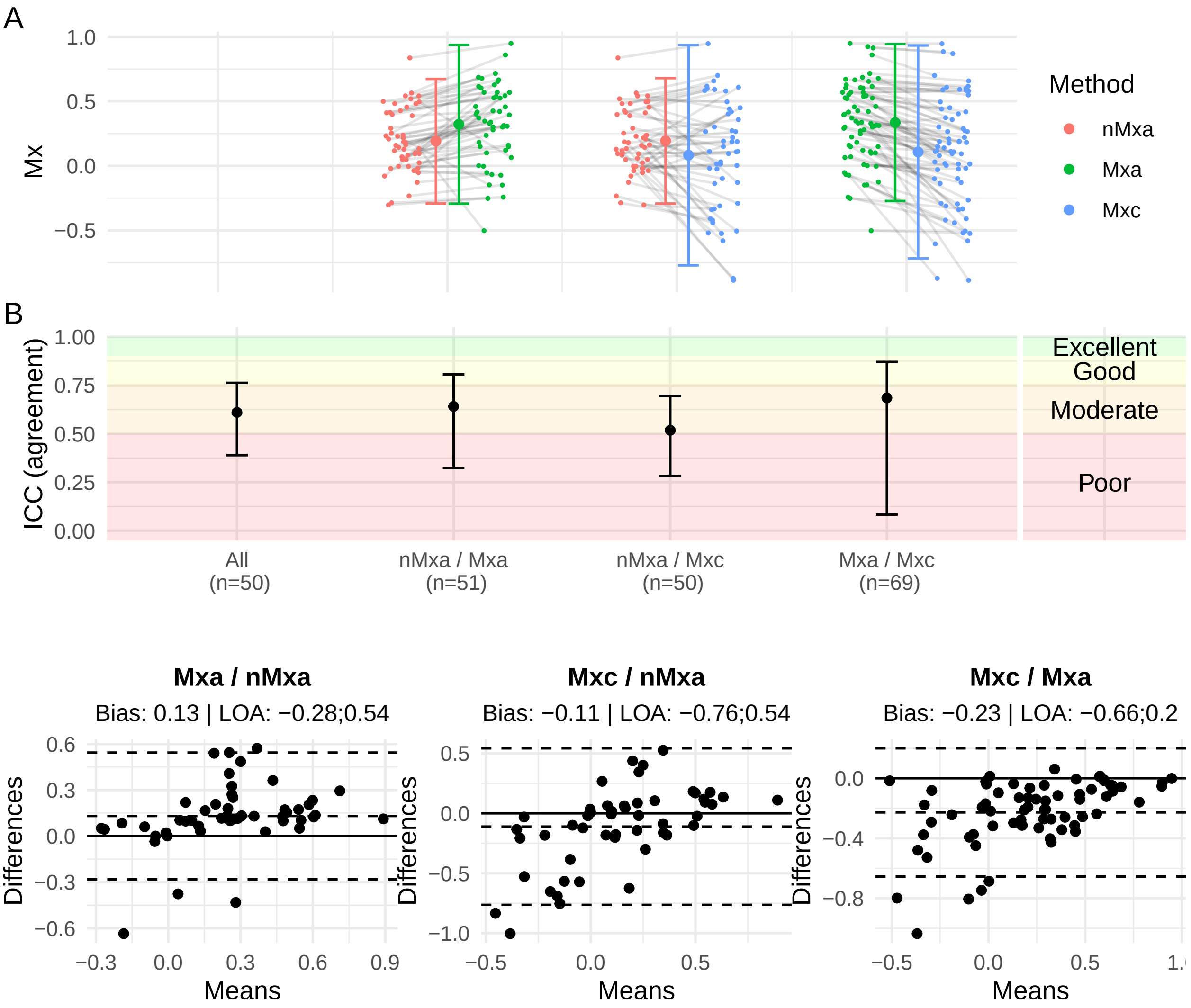

Mx analyses for only baseline-periods

Supplemental Figure 5 - Mx for induced hypertension (3-60-F)

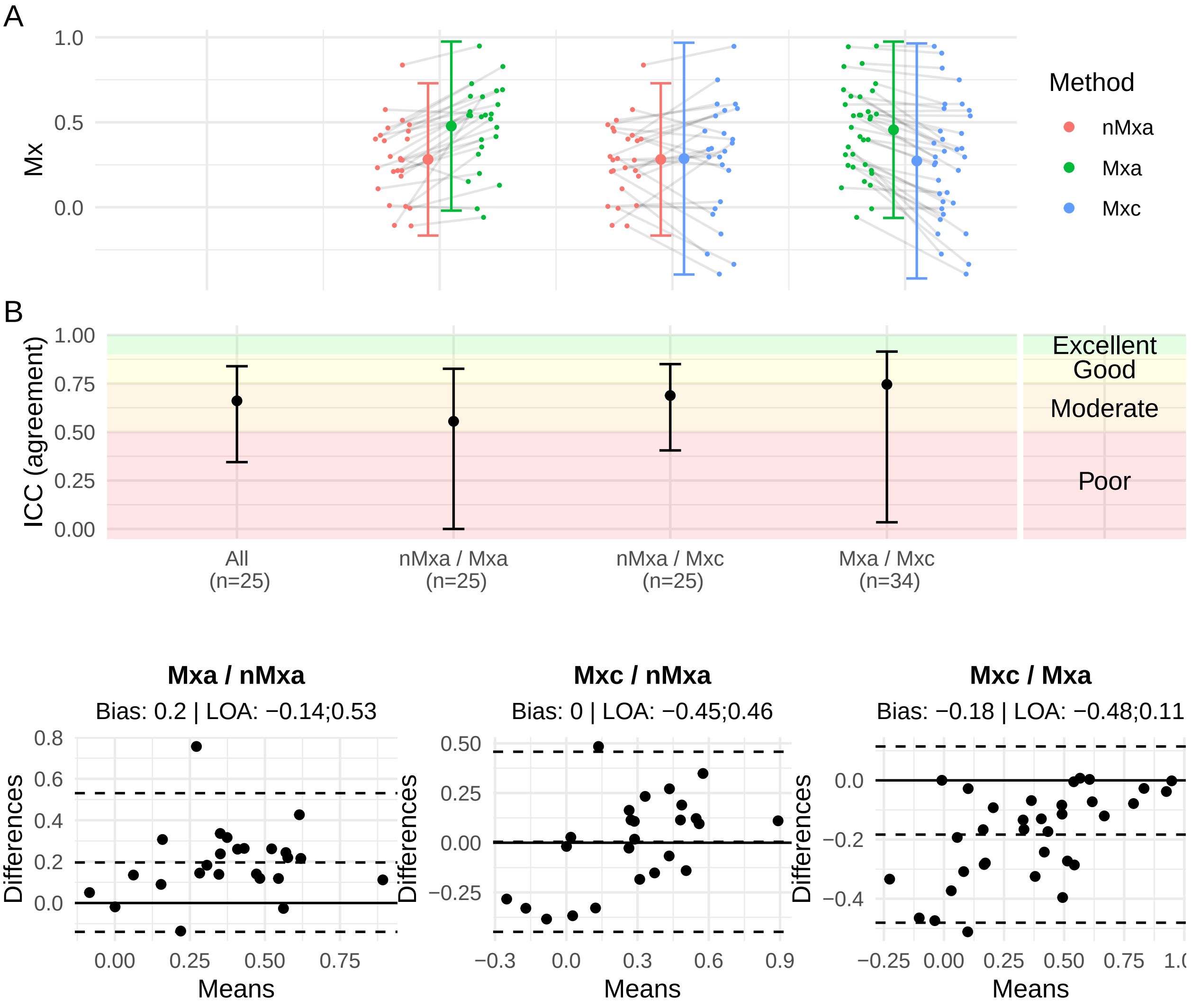

Mx analyses for only periods of induced hypertension

Bland-Altman plot for TFA

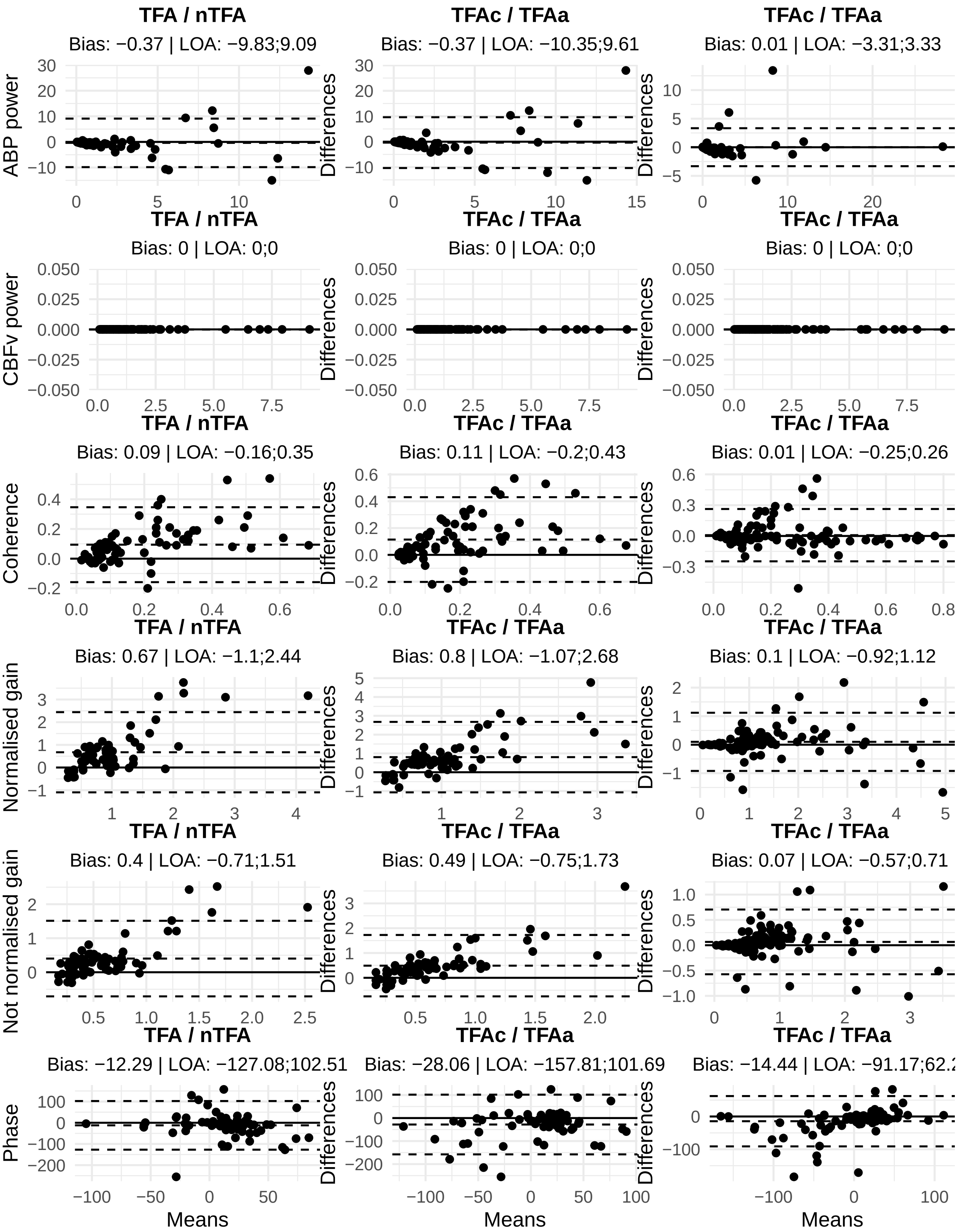

Supplement: Supplementary file 1 — Supplementary Material [file PHY2-10-e15203-s001.pdf]
